# Mitophagy in Traumatic Brain Injury: A New Target for Therapeutic Intervention

**DOI:** 10.1155/2022/4906434

**Published:** 2022-01-27

**Authors:** Mingrui Zhu, Xinqi Huang, Haiyan Shan, Mingyang Zhang

**Affiliations:** ^1^Institute of Forensic Sciences, School of Basic Medicine and Biological Sciences, Soochow University, Suzhou, China; ^2^Department of Obstetrics and Gynecology, The Affiliated Suzhou Hospital of Nanjing Medical University, Suzhou, China

## Abstract

Traumatic brain injury (TBI) contributes to death, and disability worldwide more than any other traumatic insult and damage to cellular components including mitochondria leads to the impairment of cellular functions and brain function. In neurons, mitophagy, autophagy-mediated degradation of damaged mitochondria, is a key process in cellular quality control including mitochondrial homeostasis and energy supply and plays a fundamental role in neuronal survival and health. Conversely, defective mitophagy leads to the accumulation of damaged mitochondria and cellular dysfunction, contributing to inflammation, oxidative stress, and neuronal cell death. Therefore, an extensive characterization of mitophagy-related protective mechanisms, taking into account the complex mechanisms by which each molecular player is connected to the others, may provide a rationale for the development of new therapeutic strategies in TBI patients. Here, we discuss the contribution of defective mitophagy in TBI, and the underlying molecular mechanisms of mitophagy in inflammation, oxidative stress, and neuronal cell death highlight novel therapeutics based on newly discovered mitophagy-inducing strategies.

## 1. Introduction

**Figure 1:**
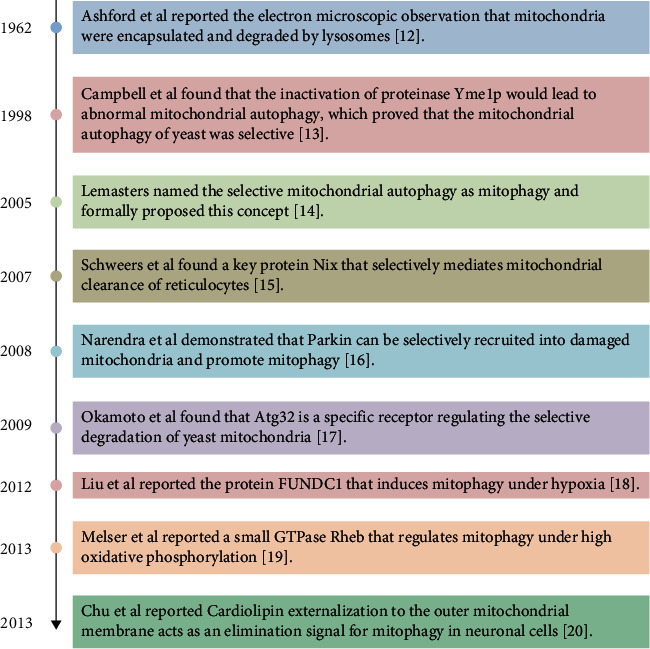
Discovery process of mitophagy [12–20].

Mitochondria are organelles coated by bilayer membranes, mitochondrial inner membrane (IMM), and mitochondrial outer membrane (OMM), which are the main places for cellular respiration and energy production [1]. The cristae, folded in the inner membrane, is a very important structure studded with ATP synthase and a variety of cytochromes [2, 3]. The brain consumes approximately 20% of the total body energy even though it accounts for only 2% of the body weight [4]. This huge energy consumption depends on the utilization of glucose and the maintenance of mitochondrial function [5]. Therefore, the maintenance of mitochondrial network integrity and activity is a prerequisite for nervous system homeostasis [6]. Defective mitochondria can be toxic by generating excessive amounts of ROS, by consuming ATP through the reversal of ATP synthase, and by interfering with a host of other metabolic processes [7]. Mitophagy is a crucial process of eliminating old or damaged mitochondria for the maintenance of the integrity of the mitochondrial pool for cellular homeostasis, which impacts various physiological and pathological courses in the brain. Recent advances in the field of brain research have revealed the pivotal role of mitophagy in neuronal cell fate and neurological function [8, 9]. However, the potential of targeting mitophagy as a therapeutic strategy in traumatic brain injury (TBI) remains to be explored. Moreover, several proteins including Cardiolipin, Parkin, and BNIP3L/NIX, which are responsible for mitophagy, have been revealed to be deregulated in TBI [10, 11]. This review is aimed at describing the research progress of mitophagy including the molecular pathways that govern mitophagy and the role of mitophagy in different aspects of TBI and other brain diseases, to develop potential therapeutics for clinical treatment (Figure 1).

## 2. The Mitophagy Pathways in TBI

**Figure 2:**
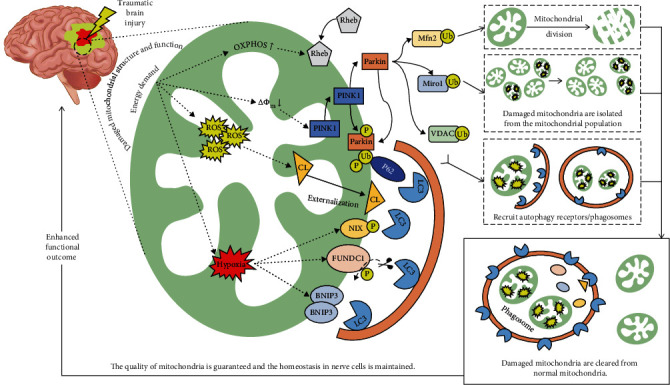
The mitophagy pathways in TBI.

Mitophagy is the targeted phagocytosis and destruction of damaged mitochondria by autophagy, whose function is to strictly control the quality and quantity of mitochondria [21]. Mitophagy is activated by a series of pathways, such as ubiquitin-mediated pathway [22] and mitochondrial receptor-mediated pathway [23, 24]. The possible mitophagy pathways in TBI are summarized in Figure 2.

Due to an energy crisis emerging post-TBI as the increased energetic demand cannot be met, the level of OXPHOS increased, and ROS levels exceeded a certain threshold. Meanwhile, hypoxia can be initiated by TBI-induced cerebral hypoperfusion. Decrease of mitochondrial membrane potential (△Ψ*m*) was also found in the brain after TBI. All the events mentioned above induce mitochondrial damage and activate different mitophagy pathways. The change in energy state caused by OXPHOS will activate Rheb pathway. Decreased mitochondrial membrane potential increases membrane depolarization and the accumulation of PINK1 kinase in the OMM. Activated PINK1/Parkin at the OMM allows the interaction with LC3 for the autophagic degradation through the specific adaptor proteins, such as p62, which binds at the same time with ubiquitin and LC3. At this time, PINK1 can recruit and activate Parkin, which regulates mitophagy by ubiquitinating Mfn2, Miro1, and VDAC. The other mechanism of mitophagy induced by stress, such as ROS and hypoxia, is regulated by mitochondrial receptors such as BNIP3, BNIP3L/NIX, FUNDC1, and Cardiolipin (CL) which directly bind with LC3 through the conserved LC3 interacting region (LIR) motif. After the above mitophagy pathways are activated, damaged mitochondria are selectively eliminated, the quality of mitochondria is guaranteed, and neuronal cells are homeostasis maintained, which improves neurological function recovery in TBI.

### 2.1. PINK1/Parkin-Mediated Ubiquitin Pathway

PTEN-induced putative kinase 1 (PINK1) is a serine/threonine protein kinase, and Parkin is a member of the E3 ubiquitin ligase complex [25]. When the mitochondrial membrane potential decreases, the expression of PINK1 is upregulated, aggregates on the mitochondrial membrane, and then, phosphorylates Ser65 in the ubiquitin-like protein domain of parkin, leading to conformational changes in the tertiary structure for the activation of parkin [26]. Activated PINK1/Parkin at the OMM allows the interaction with LC3 for autophagic degradation through specific adaptor proteins such as p62, OPTN, NDP52, and TAXBP1 which bind at the same time with ubiquitin and LC3 [16]. Our laboratory reported that the expression levels of PINK1 and Parkin were increased at 24 h after TBI and Mdivi-1 inhibited the activation of the PINK1 and Parkin pathway, suggesting that PINK1/Parkin-mediated mitophagy was activated in TBI [27]. Chen et al. demonstrated that PGAM5 deficiency blocked Parkin and PINK1 translocation to mitochondria and alleviated neuroinflammation in TBI mouse model [28]. Ren et al. found that the expression of PINK1 was significantly induced by TBI, and RvD1 treatment successfully downregulated it [29]. These results suggested that downregulation of PINK1/Parkin-mediated mitophagy might play an important role in recovery after TBI. However, Lin et al. found that PINK1 knockout mice suffering from TBI demonstrated little recovery after thyroid hormone treatment, suggesting the involvement of PINK1-mediated mitophagy in neurogenesis and neuroprotection [30]. The exact role of PINK1/Parkin-mediated mitophagy in traumatic brain injury is still unclear. The decreased expression of PINK1 and Parkin may be caused by the improvement of mitochondrial membrane potential, which is only the feedback result for mitochondrial function recovery after drug treatment and is not the reason for the improvement of brain function. PINK1/Parkin pathway alterations have also been found in many neurodegenerative diseases such as Parkinson's disease (PD), Alzheimer's disease (AD), and Huntington's disease (HD). Parkin overexpression can rescue the mitochondrial dysfunction in AD [8]. However, downregulating expression of either PINK1 or Parkin genes ameliorated neurodegeneration phenotypes in amyotrophic lateral sclerosis (ALS) [31]. Wen et al. reported that PINK1 overexpression-mediated parkin regulation is key to the protection against cerebral ischemia-reperfusion injury [32]. Therefore, we need many experiments to verify this exact role of PINK1/Parkin in TBI.

### 2.2. Receptor-Mediated Pathway

The other mechanism of mitophagy in a context-dependent manner is regulated by the mitochondrial receptors like BNIP3, BNIP3L/NIX, FUNDC1, NIPSNAP1, NIPSNAP2, BCL2L13, PHB2, FKBP8, Rheb, and Cardiolipin, which directly binds with LC3 through the conserved LC3 interacting region (LIR) motif. BCL2/adenovirus E1B 19 kDa interacting protein 3 (BNIP3), NIP3-like protein X (BNIP3L/NIX), BCL2-like 13 (BCL2L13), and FUN14 domain containing 1 (FUNDC1) are marker proteins on the OMM. They all interact with Atg8 protein family members on phagosomes through conserved LC3 interacting region (LIR) as mitochondrial receptors under hypoxia, so as to recruit phagosomes and clear mitochondria damaged by hypoxia [33]. Nitrophenylphosphatase domain and nonneuronal SNAP25-like protein homolog 1 (NIPSNAP1) and NIPSNAP2, Prohibitin 2 (PHB2), FKBP prolyl isomerase 8 (FKBP8/FKBP38) FKBP8, and Cardiolipin (CL) are located in the IMM [34]. In many cases, the IMM protein requires the rupture of the OMM to recruit the mitophagy molecular machinery. However, in some cases, this dynamic positional change of the IMM protein is not necessary for mitophagy [35]. Alternatively, they may act as direct autophagy receptors in the IMM and binds to LC3 through the classical LIR motif. There have been few reports on the role of the mitochondrial receptor-mediated mitophagy pathway in TBI. Ma et al. found that the expression of NIX decreased after TBI and NIX overexpression plays a neuroprotective role in TBI-induced damage through autophagy and apoptosis pathways [10]. In intracerebral hemorrhage and ischemia-reperfusion brain injury, NIX protein levels are significantly elevated and overexpression of NIX could be a potential therapeutic target for hemorrhagic and ischemic stroke [36, 37]. There is no doubt that hypoxia is the basic pathological mechanism in many brain diseases, including cerebral ischemic injury, TBI, and cerebral hemorrhage. However, the changes in NIX expression levels after injury in brain diseases are different, suggesting that NIX-mediated mitophagy is related not only to hypoxia but also to other pathophysiological processes, such as the cell death pathway [38, 39]. Mitochondrial lipid signaling is a hot field in TBI research. Cardiolipin (CL) is one of the unique lipids in mitochondrial intima, accounting for 15-20% of all mitochondrial lipids [40]. Kalgudi et al. found that free circulating Cardiolipins in the plasma released from the injured brain with a disrupted BBB after TBI and Cardiolipin were detectable in approximately 18% of patients after severe TBI, confirming that circulating Cardiolipin plays an important role in the pathogenesis of severe TBI in humans [41, 42]. In the TBI model, it can be observed that the selective oxidation of brain Cardiolipin begins 3 hours after injury, and enrichment and collapse of the CL were found in the IMM [11, 43]. At this time, externalized Cardiolipin can directly interact with LC3 and then promote the occurrence of mitophagy [21]. Chao et al. demonstrated that Cardiolipin-dependent mitophagy is activated by TBI, and suppression of TBI-induced mitophagy worsens the overall outcome [11]. For the Cardiolipin pathway, mitophagy can be inhibited by reducing the content of mitochondrial Cardiolipin or the expression of phospholipid scramblases (PLS). In addition, mutagenesis of amino acid residues of LC3 at R10 and R11 can reduce the ability of LC3 peptide to bind Cardiolipin in vitro and weaken the recruitment of phagosomes by LC3 in living cells [44]. Cardiolipin exposure to the outer membrane not only affects mitophagy but also modulates *α*-synuclein aggregation and affects the electron transport in PD [45]. In contrast to the above pathways, Rheb is mainly located in the OMM and matrix, which regulates mitophagy related to energy state and ensures the efficiency of mitochondrial energy production [46–48]. Our group has recently revealed that Rheb is recruited to damage mitochondria for their engulfment within mitophagosomes in the axons of neurons, and such a mechanism promotes mitophagic removal of stressed mitochondria from distal axons to maintain mitochondrial homeostasis [49, 50]. However, no reports thus far have studied the effects of mitochondrial receptors other than BNIP3L/NIX and Cardiolipin on mitophagy in TBI models. Therefore, this is an interesting aspect worth exploring.

## 3. The Role of Mitophagy in TBI

**Figure 3:**
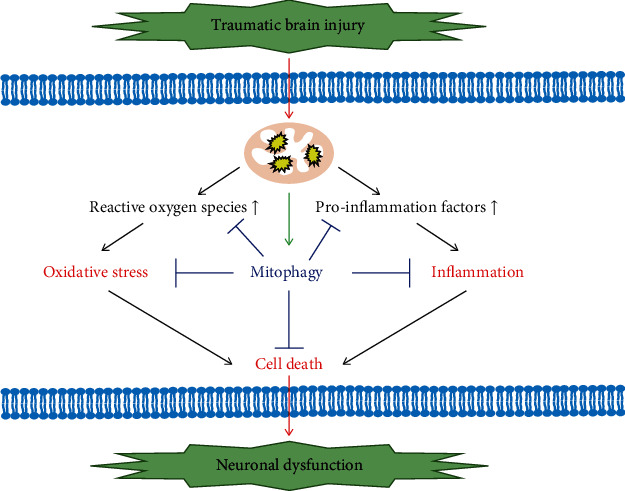
The role of mitophagy in TBI.

TBI is a traumatic structural injury and brain dysfunction caused by external forces [51], which usually originates from the primary injury caused by external causes, and then, gradually develops into secondary injury related to inflammatory response, oxidative stress, and cell death. In the occurrence and development of TBI, these biological processes interact with mitophagy to regulate mitochondrial quality, which could affect the survival and death of nerve cells (Figure 3).

Traumatic brain injury results in more severe oxidative damage, inflammatory response, and cell death, which are associated with mitochondrial dysfunction. Damage to mitochondria will release a large amount of reactive oxygen species and promote the release of proinflammatory factors, which lead to oxidative stress damage and inflammation. At the same time, damaged mitochondria result in the activation of mitophagy. If mitophagy is not enough to activate, the imbalance of the delicate equilibrium among mitophagy, ROS production, and inflammatory response can start, drive, or accelerate the cell death process. Therapeutic targets that upregulate mitophagy to reduce downstream cascades such as oxidative damage, inflammatory response, and cell death may improve neurological dysfunction after TBI.

### 3.1. Inflammation and Mitophagy

Recently, mitochondrial damage caused by TBI has gradually attracted attention, and the improvement of brain mitochondrial function has become a new target for the treatment of TBI. In the past, the main role of drugs for the treatment of TBI was to inhibit posttraumatic inflammatory response [52]. However, some drugs not only prevent inflammation induced by TBI but also improve mitochondrial function by the mitophagy pathway. Ren et al. found that Resolvin D1 not only inhibits neuroinflammation but also eliminates damaged mitochondria through mitophagy [29]. Similarly, IL-10 and p53 activators pifithrin-*μ* and pifithrin-*α* can also provide neuroprotection by regulating neuroinflammation and mitophagy [53, 54]. In addition, it was also found that rapamycin-induced mitophagy could work together with the inhibition of the activation of NLRP3-mediated inflammatory bodies, and the combined treatment had better neuroprotective effect [55]. Moreover, Lin et al. found that the inhibitory effect of melatonin on inflammation is regulated by activating mitophagy and selectively clearing damaged mitochondria. Inhibiting mitophagy will significantly enhance the inflammation caused by TBI, as direct evidence links mitophagy to inflammation [56]. Chen et al. demonstrated that PINK1-mediated mitophagy and the NLRP3 inflammasome have the interactivity, and mitophagy further enhances the neuroprotection by inhibiting NLRP3 inflammasome activation post-TBI [55]. Lin et al. highlight a role for melatonin in protecting against TBI-triggered immunopathology, which is accomplished by negatively regulating inflammation activation via mitophagy [56]. Ding et al. showed the fisetin-blocked inflammation activation via promoting mitophagy in sepsis-associated encephalopathy [57]. Zheng et al. reported that FUNDC1, as a mitophagy receptor, alleviated ICH-induced inflammation by promoting mitophagy, suggesting that FUNDC1 might be a new therapeutic target for ICH treatment [58]. Taken together, these data demonstrate that inflammation can cause accumulation of damaged mitochondria, and impaired mitophagy of these mitochondria results in the promotion of proinflammatory pathways. Thus, therapeutic targets that upregulate mitophagy to reduce downstream cascades induced by damaged mitochondria may improve inflammation.

### 3.2. Oxidative Stress and Mitophagy

During TBI, the increased energy demand leads to excessive ROS production, resulting in mitochondrial DNA damage and dysfunction [21]. Mitophagy is an endogenous neuroprotective process for targeted damaged mitochondria, and then, the damaged mitochondria can be cleared in time, which is necessary to maintain the normal function of neurons [59]. In addition, mitophagy can also reduce gastrointestinal dysfunction caused by TBI. Liu et al. found that mitophagy can reduce intestinal mucosal injury and epithelial barrier dysfunction by reducing oxidative stress after TBI [60]. However, the activation degree of mitophagy is also different due to traumatic environments and stimulation conditions. Moderate mitophagy can target damaged mitochondria, which maintains the function of mitochondria and is beneficial to the survival of neurons [61]. When mitophagy is insufficient, the damaged mitochondria are not properly cleared, and ROS continue to accumulate, resulting in the oxidation of mitochondrial proteins and DNA and the aggravation of mitochondrial damage [62]. Excessive mitophagy removes too many mitochondria [44]. Because mitochondria are crucial to neurons, excessive mitochondrial clearance will promote nerve cell death and further affect brain function [63]. Wu et al. found that mitochondrial division inhibitor 1 (Mdivi-1) can reduce TBI-induced blood-brain barrier damage and cell death by inhibiting mitophagy while inhibiting mitochondrial division [27]. In contrast, Niu et al. found that Mdivi-1 could aggravate the damage of TBI rats, indicating that mitophagy played a protective role on damaged mitochondria [64]. As noted above, the results may be caused by the degree of mitophagy. Therefore, activation of mild mitophagy or inhibition of excessive mitophagy may have beneficial effects on the structure and function of mitochondria after TBI and even on the survival of neurons [65]. In addition, it appears that mitophagy pathways differ spatially and kinetically in neurons and immortalized cells and therefore might diverge in their ultimate outcome and function. Mitophagy in nerve cells has a higher threshold than other cells and tends to local repair mechanisms or partial degradation mechanisms [44]. In 2020, Zakarya et al. found that 5,6-dicarboxy-1,1,3,3-tetraethylisoindolin-2-yloxyl (DCTEIO), as a superoxide dismutase simulant, can maintain the structure and function of mitochondria by scavenging ROS, reduce oxidative stress, promote mitophagy, and then, improve the tissue repair and neural function of TBI rats. Animal experiments also showed that an obvious increase in mitophagy, and the damage range of brain tissue decreased at 24 hours and 6 weeks after injury when treated with DCTEIO immediately after injury [66]. Lin et al. demonstrated that triiodothyronine (T3) promoted the clearance of damaged mitochondria by promoting PINK1 and significantly reduced the production of ROS, indicating that T3 reduced neuronal death through mitophagy [30]. These results indicate that mitophagy prevents the accumulation of damaged mitochondria and ROS.

### 3.3. Cell Death and Mitophagy

Cell death is the final solution for a neuron only when multiple stresses are piled up to a level beyond cell's recovery capacity [67]. A traumatic incident, such as TBI, causes a sudden decline of energy production in the affected neurons and elicits acute neuronal cell death, which is commonly seen in neurodegenerative diseases. Two waves of neuronal cell death, necrosis, and programmed cell death occur after TBI. Early application of neuroprotective protocols seems critical for any possibility of reducing neuronal necrosis induced by membrane disruption and irreversible metabolic disturbances. This second wave of neuronal programmed cell death presents within a time window that may be responsive to targeted therapies [68, 69]. Recent studies have shown that in addition to necrosis, there are also other new programmed death modes, such as apoptosis, necroptosis, pyroptosis, ferroptosis, entotic cell death, netotic cell death, parthanatos, lysosome-dependent cell death, autophagic cell death, alkaliptosis, and oxeiptosis [70]. Mitophagy, the removal of damaged or unwanted mitochondria, was found to be essential for maintaining cellular fitness [71]. Previous work by our group demonstrated the role of mitophagy in regulating cell death [72]. The current review summarizes the relationship between mitophagy and cell death after TBI, as well as mitophagy as a potential therapeutic intervention for altering cell death to improve neurological outcomes. Apoptosis is a type of programmed cell death (PCD). The cytomorphological features of apoptotic cells include shrinkage, chromosome condensation, and DNA fragmentation [67]. Niu et al. demonstrated that Mdivi-1 blocked the induction of mitophagy specifically and activated the apoptosis markers caspase-3 and caspase-9, implying that mitophagy markedly decreased cell apoptosis induced by TBI [64]. Wu et al. reported that Mdivi-1 also alleviated the number of LC3 puncta and TUNEL-positive structures in cells, indicating that autophagy may be involved in the antiapoptotic effects of Mdivi-1 in TBI [27]. Chao et al. showed that TBI-induced Cardiolipin-dependent mitophagy is an endogenous neuroprotective process that limits neuronal apoptosis and behavioral deficits [11]. Lin et al. found that T3 treatment could provide a therapeutic approach for TBI by preventing apoptosis via pink1-mediated mitophagy [30]. Pyroptosis represents a form of programmed cell death that is triggered by proinflammatory signals and associated with inflammation [73]. Recent studies have shown that oxygen deprivation or hypercapnia promotes microglial pyroptosis by inhibiting mitophagy, suggesting that mitophagy acts as a negative regulator of pyroptosis [74, 75]. Chen et al. demonstrated that rapamycin-induced mitophagy enhances the neuroprotection of inhibition of pyroptosis activation post-TBI [55]. Quercetin also prevents neuronal injury via inhibition of pyroptosis activation in microglia by promoting mitophagy in both depression and PD mouse models [76]. Ferroptosis is a new type of cell death that was discovered in recent years and is usually accompanied by a large amount of iron accumulation and lipid peroxidation during the cell death process [77]. It has been reported that ferritin deficiency promotes osteoblastic ferroptosis via the PINK1/Parkin-mediated mitophagy pathway in type 2 diabetic osteoporosis [78]. However, there is no report about the relationship between mitophagy and other cell deaths including ferroptosis in the brain, and a deeper understanding of the relationship between ferroptosis and multiple diseases may provide new strategies for drug development based on ferroptosis.

### 3.4. Related Therapeutic Agents Targeting Mitophagy in TBI

Therapeutic targets that upregulate mitophagy to reduce downstream cascades such as oxidative damage, inflammatory response, and cell death may improve neurological dysfunction after TBI. Therefore, we elucidate the mitophagy activators and inhibitors used in TBI in this section (Table 1).

## 4. Conclusion

In recent years, extensive efforts have been made to discover and develop new drugs targeting mitochondrial dysfunction to efficiently rescue or ameliorate the outcomes of traumatic brain injury. In neurons, efficient clearance of damaged mitochondria through mitophagy plays a fundamental role in mitochondrial and metabolic homeostasis, energy supply, neuronal survival, and health. The brain functional outcome was correlated with the severity of the injury after TBI. Therefore, the type, extent, and spatiotemporal distribution of neuronal mitophagy could be related to injury type and severity. Therefore, mitophagy stimulation might be both beneficial and detrimental for tissue homeostasis depending on cellular bioenergetics under certain pathophysiological conditions. Despite the extensive studies focusing on the molecular mechanisms that govern mitophagy, some outstanding questions remain. First, it is necessary to further study the interconnected interactions between the different mitophagy pathways and how they coordinate the regulation of mitochondrial removal. Second, the multifaceted roles of mitophagy in cell survival and death are needed to be further investigated. Last, it is necessary to further develop specific, robust, but low-toxicity mitophagy inducers for clinical benefits.  Table 2 shows the summary of abbreviations.

## Figures and Tables

**Table 1:** Summary of therapeutic development targeting mitophagy in TBI.

Methods or compounds	Time	Effect on mitophagy	Doses	Action site	Function in TBI	Reference
Resolvin D1	2020	Activate	15 *μ*g/kg	Inflammation	Ameliorate brain oedema and cognitive impairment, suppress neuroinflammation and neuronal loss，eliminate extra mitoROS, improve the supportive function of astrocytes	[29]
IL-10	2019	Activate	Overexpress	Inflammation	Inhibit inflammatory response, reduce neuronal degeneration and death	[53]
Pifithrin-*μ* Pifithrin-*α*	2019	Mitigate	2 mg/kg	Inflammation	Ameliorate neurological functional deficits, attenuate neuroinflammation, attenuate oxidative stress	[54]
Rapamycin	2019	Activate	3 mg/kg	Inflammation Cell death	Attenuate neuroinflammation, mitochondrial damage, demonstrate neuroprotective effects, inhibit the activation of NLRP3 inflammasome	[55]
Melatonin	2016	Activate	5 ml/kg	Inflammation	Repress inflammation, ameliorate neuronal death and behavioral deficits, dampen the secretion of pro-inflammatory cytokines	[56]
Rapamycin	2016	Activate	/	Oxidative stress	Alleviate TBI-induced intestinal mucosa damage and epithelial barrier dysfunction	[60]
Mdivi-1	2017	Suppress	3 mg/kg	Oxidative stress Cell death	Alleviate loss of mitochondrial membrane potential, ROS production, ATP reduction, blood-brain barrier disruption and cell death	[27]
Mdivi-1	2018	Suppress	1 mg/kg	Oxidative stress Cell death	Aggravate neurological manifestations and neuronal apoptosis	[64]
DCTEIO	2020	Activate	40 mM aqueous solutions	Oxidative stress	Scavenge ROS, improve tissue repair and preserve neurological function	[66]
Triiodothyronine	2020	Activate	20 *μ*g/100 g	Oxidative stress Cell death	Reduce ROS production, prevent neuronal death, induce neurogenesis and neuroprotection	[30]
Cardiolipin siRNA	2019	Activate	30 nmol	Cell death	Induce endogenous neuroprotection, limit neuronal apoptosis and behavioral deficits	[11]

**Table 2:** Summary of abbreviations.

Abbreviations	Full name
IMM	Inner membrane of mitochondrial
OMM	Outer membrane of mitochondrial
ATP	Adenosine triphosphate
ADP	Adenosine diphosphate
OXPHOS	Oxidative phosphorylation
ROS	Reactive oxygen species
TBI	Traumatic brain injury
SOD	Superoxide dismutase
PINK1	PTEN-induced putative kinase 1
NRF2	Nuclear factor-erythroid 2-related factor 2
KEAP1	Kelch-like ECH-associated protein 1
TOM	Translocase of the outer membrane
OMS	Outer mitochondrial membrane localization signal
MFN	Mitochondrial fusion
Miro1	Mitochondrial Rho GTPase 1
VDAC	Voltage-dependent anion channel
LIR	LC3 interaction region
LC3	Microtubule-associated protein 1 light chain 3
NIX	NIP3-like protein X
Bnip3	BCL2/adenovirus E1B 19 kDa interacting protein 3
FUNDC1	FUN14 domain containing 1
OPA1	Optic atrophy 1
Ser	Serine
Tyr	Tyrosine
CK2	Casein kinase 2
PGAM5	Phosphoglycerate mutase family member 5
ULK1	The yeast ATG1 homologues
CL	Cardiolipin
PLS	Phospholipid scramblase
Thr	Threonine
Mdivi-1	Mitochondrial division inhibitor 1
DCTEIO	5,6-Dicarboxy-1,1,3,3-tetraethylisoindolin-2-yloxyl
T3	Triiodothyronine
NDP52	Nuclear dot protein 52 kDa
TAXBP1	Tax1-binding protein 1
NIPSNAP1/2	Nitrophenylphosphatase domain and 147 nonneuronal SNAP25-like protein homolog 1/2
PHB2	Prohibitin 2
FKBP8	FKBP Prolyl Isomerase 8
